# Chlorophyll catalyse the photo-transformation of carcinogenic benzo[a]pyrene in water

**DOI:** 10.1038/srep12776

**Published:** 2015-08-04

**Authors:** Lijuan Luo, Xueying Lai, Baowei Chen, Li Lin, Ling Fang, Nora F. Y. Tam, Tiangang Luan

**Affiliations:** 1 MOE Key Laboratory of Aquatic Product Safety, South China Sea Bio-Resource Exploitation and Utilization Collaborative Innovation Center, School of Life Sciences/School of Marine Science, Sun Yat-sen University, Guangzhou 510275, China; 2 Instrumental Analysis and Research Center, Sun Yat-Sen University, Guangzhou 510275, China; 3 Department of Biology and Chemistry, City University of Hong Kong, Tat Chee Avenue, Kowloon, Hong Kong SAR, China.

## Abstract

Algal blooms cause great damage to water quality and aquaculture. However, this study showed that dead algal cells and chlorophyll could accelerate the photo-transformation of benzo[a]pyrene (BaP), a ubiquitous and persistent pollutant with potently mutagenic and carcinogenic toxicities, under visible light irradiation. Chlorophyll was found to be the major active substance in dead algal cells, and generated a high level of singlet oxygen to catalyse the photo-transformation of BaP. According to various BaP metabolites formed, the degradation mechanism was proposed as that chlorophyll in dead algal cells photo-oxidized BaP to quinones via photocatalytic generation of singlet oxygen. The results provided a good insight into the role of chlorophyll in the photo-transformation of organic contaminants and could be a possible remediation strategy of organic pollutants in natural environment.

Benzo[a]pyrene (BaP), one of the polycyclic aromatic hydrocarbons (PAHs) containing five fused benzene rings, is an ubiquitous pollutant with potently mutagenic and carcinogenic toxicities^1^. It has been ranked as the first class of “human carcinogens” in the report of World Health Organization (WHO) International Agency for Research on Cancer^2^. BaP is persistent in the environment due to its high *K*_*ow*_ and low vapour pressure, and also has a strong sorption to organic matter in soils and a low bioavailability. It is widespread in the air, water and soil, and the level ranges from not detected to 84 ng L^−1^ in the water, from 4.7 to 288.7 ng g^−1^ in the suspended particulate matters and reaches up to 47.9 ng g^−1^ in the sediment^3^,^4^,^5^. BaP is a well-studied member of the PAH family and serves as a model compound for understanding the degradation and carcinogenic effects of PAHs^6^.

Chemical oxidation, photo-oxidation, microbiological degradation and bioaccumulation are the main methods utilized to eliminate BaP from the environment^7^. Bioremediation by microorganisms has been suggested as an attractive means^6^. It is more effective and economical than chemical oxidation and photo-oxidation. Recently, microbial degradation of BaP has mainly focused on bacteria and fungi whereas less attention has been paid on microalgae^8^,^9^,^10^. Even so, the role of microalgae could not be ignored because microalgae are prevalent in various aquatic habitants worldwide. *Selenastrum capricornutum*, a freshwater microalga, was demonstrated to have the capacity of degrading BaP^11^,^12^. In comparison to live microalgal cells, dead cells of *S. capricornutum* exhibited high removal rates of high molecular weight (HMW) PAHs, including benz[a]anthracene, BaP, dibenzo[a,h]anthracene, indeno[1,2,3-c,d]pyrene and benzo[g,h,i]perylene, and dead cells also had greater transformation abilities than live cells under white light irradiation^13^. The transformation of PAHs in live algal cells was closely relied on the occurrence and activity of intracellular enzymes^14^. On the other hand, dead cells with the unique enzyme systems being inactivated possibly acted as a photosensitizer to stimulate the photo-degradation of PAHs^13^.

Some studies have reported that dead algal cells could accelerate the photolysis of contaminants under light irradiation, such as pesticides^15^, aniline^16^ and bisphenol A^17^. However, the mechanism responsible for accelerating PAH transformation by dead algal cells remains to be elucidated. It is possible that cellular components releasing from dead algal cells could catalyse photo-reactions with PAHs. However, it is still unknown what component or which group of components exactly play a key role in the degradation of PAHs by dead cells.

The photo-degradation of PAHs in aqueous solution was generally related to reactive oxygen species (ROS), including hydroxyl radical (•OH), singlet oxygen (^1^O_2_), hydrogen peroxide (H_2_O_2_) and superoxide radical (O_2_^-^)^17^,^18^,^19^,^20^. In previous studies, •OH or ^1^O_2_ was speculated to play crucial roles in the photo-degradation of organic pollutants^21^,^22^,^23^,^24^. Few studies focused on the relationship of algae and ROS during the photo-degradation process. For instance, Zepp *et al.*^25^ found that several algae (*Chlamydomonas* sp., *Chlorogonium* sp. and *Anaebaena variabilis*) induced the photoproduction of H_2_O_2_ in the oxidation of anilines. •OH could be induced by microalgae of *Nitzschia hantzschiana*, *C. vulgaris* and *Anabaena cylindrica* in the aqueous solution under high-pressure mercury lamp^26^. Chlorophyll is abundant in microalgal cells, and there are clear evidences that chlorophyll is endowed with photosensitizer properties, mediates ROS generation under light irradiation^27^. Hence chlorophyll might be involved in BaP photo-transformation. However, the exact role of chlorophyll in the photo-transformation of organic pollutants in the water has never been reported.

The major objective of this study was to elucidate the mechanism of BaP photo-transformation induced by dead algal cells in the water. In our previous studies, dead algal cells were shown to be effective in BaP transformation under light irradiation^13^,^28^. Cellular contents releasing from dead algal cells, especially chlorophyll extracted from microalgae, were employed to test the photo-transformation rate of BaP under visible light irradiation. Effects of various ROS (•OH and ^1^O_2_) on the photo-transformation of BaP were also investigated.

## Results

### Photo-transformation of BaP by dead microalgal cells

**Figure 1:**
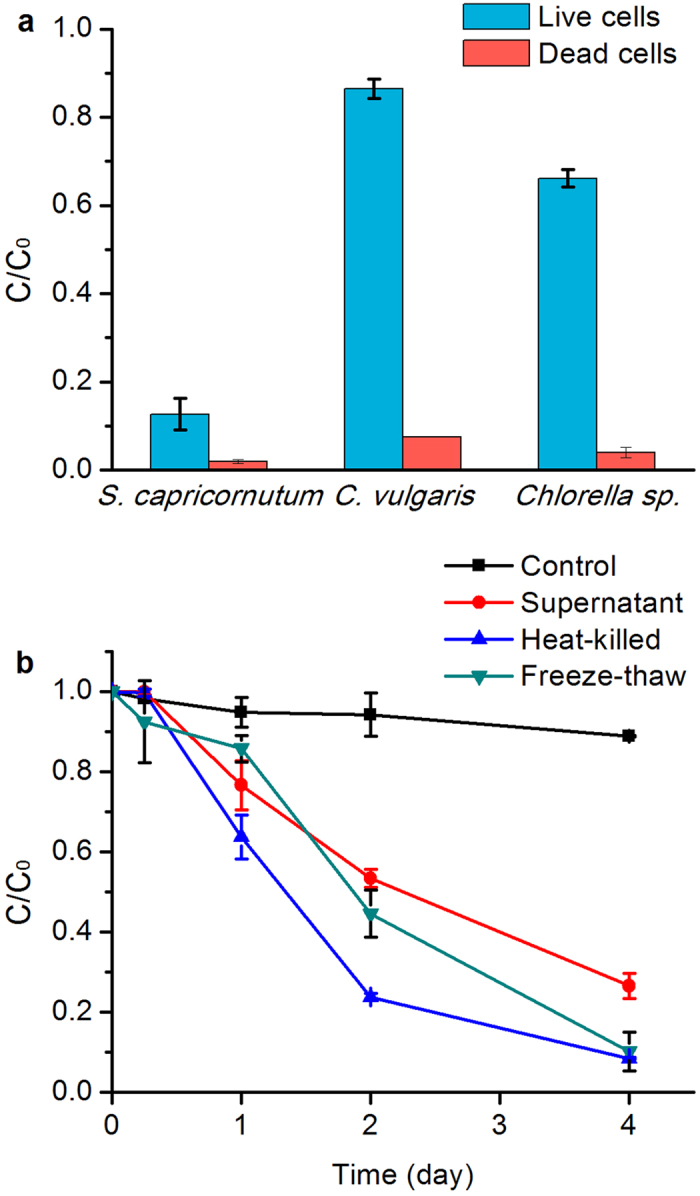
Photo-transformation of BaP by dead microalgal cells. (**a**) Effects of algal species by live or dead cells of *S. capricornutum*, *C. vulgaris* and *Chlorella* sp. under white light irradiation at Day 4. (**b**) Effects of algal cell inactivated methods and supernatant of dead *S. capricornutum*. Mean ± SD, n = 3.

In order to confirm the efficiency of dead algal cells in BaP transformation, three different microalgal species, namely *S. capricornutum*, *Chlorella vulgaris* and *Chlorella* sp., were tested (Fig. 1a). Among live algal cells, *S. capricornutum* had the highest degradation efficiency, with approximately 87% degradation of BaP after 4 days. In comparison, only 13.6% of BaP was degraded by *C. vulgaris*, the species exhibited the lowest degradation efficiency. These revealed that BaP biotransformation in live algal cells was species-dependent. No significant differences in the transformation efficiency of Bap were observed among the dead algal cells, and approximately 98.1, 92.5 and 96.0% of BaP were transformed by *S. capricornutum*, *C. vulgaris* and *Chlorella* sp., respectively. This result suggested that BaP transformation by dead cells was higher than live cells and was independent of algal species.

Freezing-thawing method can sacrifice microalgae while the activities of enzymes are maintained^29^. Thus two different preparation approaches of dead algal cells (heat-killing and freezing-thawing) were used to investigate their potential effects on the photo-transformation of BaP. The BaP photo-transformation rate in the initial 3 days was lower in freezing-thawing cells than in heat-killing cells, but the photo-transformation efficiencies on the 4^th^ day were comparable (Fig. 1b). These findings not only indicated that the final rate of BaP photo-transformation by dead algal cells was not significantly affected by the preparation method of dead cells, they also suggested that microalgal enzymes were not important in the photo-transformation of BaP. The supernatant of cell lysate followed similar trends as dead cells, with an increasing transformation ratio of BaP over time, from 18.2% (Day 1) to 40.8% (Day 2), and to 62.2% on Day 4 (Fig. 1b). It indicated that the components in the supernatant fraction also accelerated the photo-transformation of BaP.

### Photo-transformation of BaP by chlorophyll

**Figure 2:**
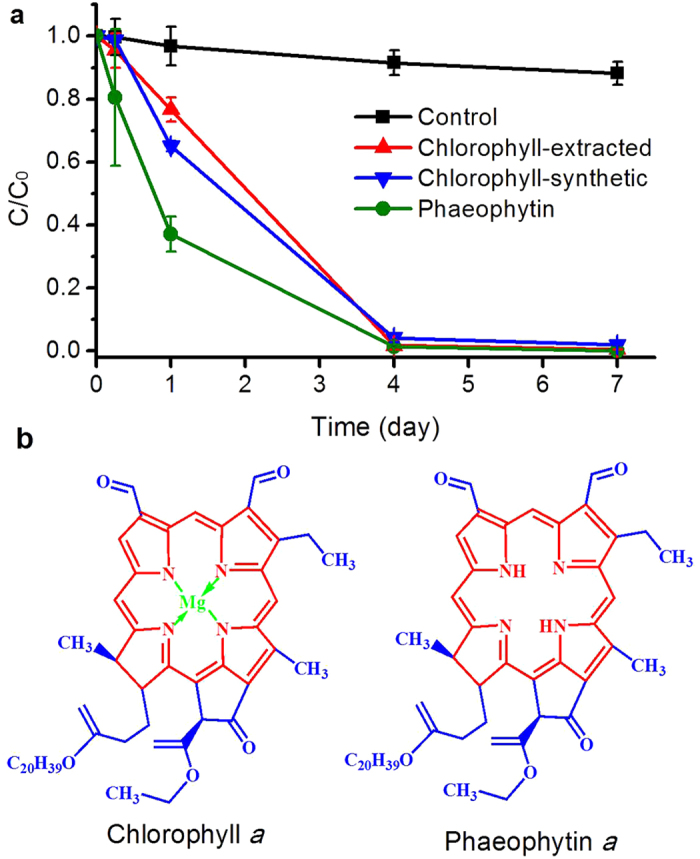
Chlorophyll catalysed the photo-transformation of BaP under light irradiation. (**a**) Effect of chlorophyll extracted from *S. capricornutum*, synthetic chlorophyll *a* and phaeophytin on BaP photo-transformation. Mean ± SD, n = 3. (**b**) Chemical structures of chlorophyll *a* and phaeophytin *a*.

Chlorophyll can absorb light energy for photosynthesis and is also abundant in green algae. It was therefore hypothesized that chlorophyll might play a key role in the photo-transformation of BaP. Chlorophyll was extracted from *S. capricornutum* to investigate its role in the photo-transformation of BaP. In the cells of *S. capricornutum*, chlorophyll is comprised of chlorophyll *a* and *b*, and they differ only in the composition of a side chain (in *a* it is -CH_3_ and in *b* it is CHO). Chlorophyll *a* accounted for approximately 88% of the total chlorophyll in *S. capricornutum*^30^. So chlorophyll *a* was substituted for chlorophyll. The chlorophyll *a* concentration of *S. capricornutum* at a density of 3.5 × 10^6^ cell mL^−1^ was 1.1 μg mL^−1^. So the concentration of chlorophyll utilized in the assays was set at the same value. The residual amount of BaP plummeted from Hour 6 to Day 4, and at least 98.2% of BaP was photo-oxidized accordingly after 4-days irradiation (Fig. 2a). The photo-transformation of BaP was also carried out with synthetic chlorophyll *a* at the same time under the same condition. The results were similar to the chlorophyll extracted directly from algal cells (Fig. 2a). As previous study reported that chlorophyll could be converted into phaeophytin under high temperature and light irradiation^31^, the effect of phaeophytin on BaP transformation was also examined. Phaeophytin transformed BaP at a rate faster than chlorophyll in the initial 3 days, but the photo-transformation efficiencies were comparable after 4 days of irradiation, with a total of 98.5% of BaP being photo-oxidized in all treatments from Day 4 onwards (Fig. 2a). All findings corroborated that chlorophyll in the algal cell lysate was the major active substance accelerating the photo-transformation of BaP under light irradiation.

The effect of chlorophyll *a* concentration on the BaP photo-transformation is shown in Supplementary Fig. 1. No photo-transformation of BaP was observed at the concentration of chlorophyll as low as 0.1 μg mL^−1^, but significant amount of BaP was transformed at a concentration of 1.0 μg mL^−1^.

### Photo-production of ^1^O_2_ and •OH

**Figure 3:**
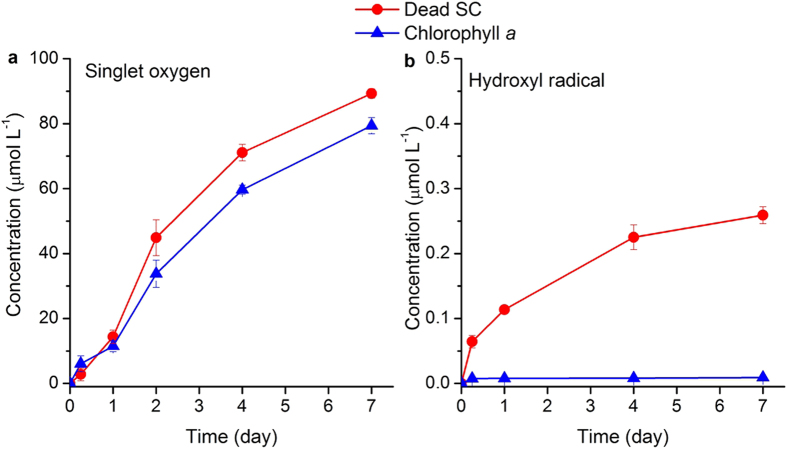
Photo-production of (**a**) singlet oxygen and (**b**) hydroxyl radical in the suspension of dead algal cells and chlorophyll extracted from *S. capricornutum* under white light irradiation. SC, *S. capricornutum*. Mean ± SD, n = 3.

The photochemical-generated •OH and ^1^O_2_ are both capable of reacting with PAHs^20^. The levels of ^1^O_2_ and •OH in the aqueous solution under light irradiation were measured, where furfuryl alcohol (FFA) and benzene were used as trapping agents to determine the levels of ^1^O_2_ and •OH, respectively. Figure 3 shows that dead algal cells and chlorophyll could generate both ^1^O_2_ and •OH under visible light irradiation. The generation rate of ^1^O_2_ was much higher than that of •OH (Supplementary Table 1), implying that ^1^O_2_ could be a primary driver for BaP photo-transformation.

The photosensitized reaction can be described by first-order rate equation^20^:


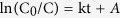


The first-order rate constants k for BaP photo-transformation in dead algal cells and chlorophyll were calculated from the linear regression In (C_0_/C) vs. time (t) with all regression coefficients more than 0.9 and are shown in Supplementary Table 2. The BaP photo-transformation rate was much lower than ^1^O_2_ generation rate in the presence of dead *S. capricornutum*, indicating that the oxidation of BaP instead of the photo-production of ^1^O_2_ was a limiting step for the BaP transformation. This also implied that the transformation rate of BaP in dead algal cells could be enhanced by increasing the oxidation rate of BaP with ^1^O_2_.

### BaP metabolites in microalgae and chlorophyll

**Figure 4:**
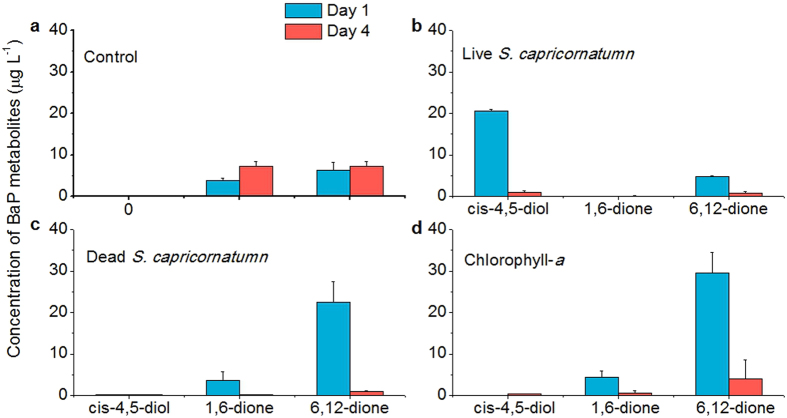
Concentrations of BaP metabolites. (**a**) Controls, (**b**) and (**c**) live and dead algal cells of *S. capricornutum* and (**d**) chlorophyll *a* extracted from *S. capricornutum*. The initial concentration of BaP was 100 μg L^−1^. Control, blank culture medium; *cis*-4,5-diol, BaP-*cis*-4,5-dihydrodiol; 1,6-dione, BaP-1,6-dione; 6,12-dione, BaP-6,12-dione. Mean ± SD, n = 3

Identifying transformation products could provide key insights into the reaction pathways and mechanisms of PAH photo-transformation by dead microbial cells and chlorophyll. The metabolites of BaP were identified using LC-APCI-MS, and the results are shown in Fig. 4. BaP-1,6-dione and BaP-6,12-dione could be detected in the controls without algal cells, corresponding to the abiotic loss of BaP in control flasks (~10%, Fig. 1b). The peak between BaP-1,6 and -6,12-dione (retention time of 3.05 min) was identified as Bap-3,6-dione according to the characteristic molecular ions (see Supplementary Table 2) and the typical mass spectrum reported in the literature^32^. Previous studies have shown BaP-1,6, -3,6, and -6,12-dione are the primary photo-transformation products of BaP under visible irradiation^33^.

In the treatment with live *S. capricornutum*, BaP was metabolized into BaP-*cis*-4,5-diol and quinones, and the former was identified as the major metabolite. This meant that biotransformation and photo-transformation of BaP occurred simultaneously in the treatment of live cells, but biotransformation was predominant. On the contrary, in the treatments of dead *S. capricornutum* cells and chlorophyll, BaP quinones were predominant over other metabolites and the production of the quinones was higher than those of live algal cells. The concentrations of BaP metabolites in all treatments except for controls decreased over time.

## Discussion

BaP biotransformation by live algal cells was species-dependent, probably due to significant differences in the enzyme system of each species, such as o-diphenol oxidase, cytochrome P450 and peroxidase^14^. The photo-transformation of BaP by dead algal cells was species-independent, since the unique enzymes relating to BaP transformation were probably inactivated partially or completely. It is a great advantage to utilize dead algal cells to eliminate pollutants from natural environment. First, it is easier to handle “dead” than “live” microalgae, particularly in wastewater treatment, as dead cells do not need any supplementary growth requirements such as energy and nutrients. Second, “dead” microalgae were not affected by any toxic pollutants in wastewater, therefore they are more applicable in treating different types of wastewater, including those containing toxic pollutants such as PAHs and heavy metals. Third, release of “live” microalgae may result in excessive production of chlorophyll leading to algal blooms in natural aquatic environments. According to Fig. 1b, BaP photo-transformation efficiency was not significantly influenced by the preparation method of dead cells after 4 days irradiation, suggesting the algal cells could accelerate the transformation of BaP irrespective to whether they were artificially inactivated or naturally killed.

Besides dead algal cells, the supernatant of cell lysate also led to an increasing transformation ratio of BaP (Fig. 1b). A similar phenomenon was found in the photolysis of aniline, and the photolysis rate of aniline under sunlight irradiation was higher in the supernatant of dead algal cells than that in distilled water^16^. Some researchers were very interested in these findings and attempted to find out the substance in dead algal cells catalysing the photolysis of organic pollutants. Wang *et al.*^18^ used Fourier-Transform Infrared (FT-IR) spectroscopy to qualify the algal exudates in the supernatants of dead algal cells, and the results showed that the compounds containing carboxylic acids were the major constitute. Carboxylic acid-containing compounds might be formed from the lipid compounds released from heat-killed algal cells^34^. Some coloured organic complexes such as humic and fulvic acids were proposed as photosensitizers in the photo-oxidative reactions^17^. However, they are not the light-sensitive substances, and there was no direct evidence to substantiate the relationship between the photo-degradation of organic pollutants and the above mentioned biomolecules.

In this study, chlorophyll was corroborated the major active substance accelerating the photo-transformation of BaP under light irradiation. Nearly 100% of BaP was degraded in the solution of chlorophyll, either the chlorophyll extracted from *S. capricornutum* or synthetic chlorophyll *a* (Fig. 2a). Many researchers demonstrated that dissolved organic matter (DOM) exerted a significant influence on the photo-transformation of organic contaminants in the natural water^35^,^36^, but the role of chlorophyll in enhancing the photo-transformation of BaP has never been reported. Chlorophyll is essentially comprised of a substituted porphyrin ring and phytol (the long carbon chain), and an Mg atom at the centre of porphyrin ring is involved in absorbing light energy (Fig. 2b). Due to the porphyrin core structure, chlorophyll exhibits a high photo-activity. Porphyrins have been proved to act as an efficient photosensitizer for the photo-transformation of other organic compounds, such as pesticides^37^, 4-nitrophenol^38^, and dye^39^,^40^. Chlorophyll was employed as a template to prepare molecularly imprinted polymers for the separation of photoactive porphyrin-like substances^41^. Besides chlorophyll, phaeophytin also had the capacity of photooxidation of BaP, with 98.5% of BaP transformation after 4 days irradiation in this study. Phaeophytin transformed BaP at a rate faster than chlorophyll, especially in the initial of irradiation (Fig. 2a), which was consistent with the result that the photo-transformation rate of BaP in heat-killing cells was higher than in freezing-thawing cells in the first three days (Fig. 1b). During heat killing process, high temperature could change the chemical structure of chlorophyll, the central Mg atom of the porphyrin ring could be removed and chlorophyll was converted into phaeophytin (Fig. 2b). Previous study also reported the formation of phaeophytin during chlorophyll degradation^31^. The structure of phaeophytin was similar to chlorophyll, and porphyrin ring might play an important role in enhancing the photo-transformation of BaP. As the structures of chlorophyll and phaeophytin are unstable under light irradiation^42^, the role of degradation products and derivatives of chlorophyll in the photo-degradation of organic pollutants deserved further studies.

The reason for chlorophyll to accelerate the photo-transformation of BaP is that chlorophyll has the photosensitizer property to generate ^1^O_2_^27^. After absorbing light energy, chlorophyll reaches triplet state, energy is transferred to the ground state oxygen and results in the formation of ^1^O_2_ by producing spin reversal of one electron in O_2_^43^. The high level of ^1^O_2_ favored the formation of BaP quinones which were the predominant metabolites in the present of dead algal cells and chlorophyll (Figs 3a and 4). BaP quinones could be produced by attacking of ^1^O_2_ on three sites of BaP, including K region, bay region and 6-position^33^,^44^,^45^. The photo-transformation efficiency of BaP was found increased with the concentrations of chlorophyll-*a* (Supplementary Fig. 1), probably due to the increased generation of ^1^O_2_ with the concentrations of chlorophyll. At low chlorophyll concentration (0.1 μg mL^−1^), the amount of singlet oxygen generated was too small to cause significant degradation of BaP.

Under light irradiation, BaP could also result in the generation of ^1^O_2_ which could be quickly consumed by BaP oxidation^35^. The photo-production of ^1^O_2_ by BaP was slow, and consequently 11.8% of BaP was converted into quinones over 7 days in the control (Supplementary Table 1, Fig. 4a). However, the ^1^O_2_ generation was fast in the presence of chlorophyll and could reach a rate of 11.67 μmol L^−1^ d^−1^ (Supplementary Table 1), and almost all of BaP was eliminated at Day 4 (Fig. 2a). The absence of monohydroxyl BaP was in good agreement with the low production of •OH that is essential to the generation of monohydroxyl BaP (Figs 3b and 4).

Dihydroxyl BaP was the major metabolite and monohydroxyl BaP was not found in the treatments of live algal cells (Fig. 4b), which was in good accordance to previous results that only a small amount of BaP (only 2%) was transformed into monohydroxyl BaP by live cells of *S. capricornutum*^46^, and the microalgae metabolizes BaP to *cis*-dihydrodiols preferentially via the dioxygenation route instead of monooxygenation^11^,^46^. The concentrations of BaP metabolites in all treatments except controls decreased over time, similar declining trends were also reported by Olmos-Espejel and co-workers^47^ on BaP-*cis*-4,5-diol. It might be ascribed to the conjugation of BaP metabolites by *S. capricornutum* (71% of BaP metabolites), 12.2%. 12.0% and 12.4% of BaP metabolites were conjugated with sulfate ester, α and β-glucose conjugates, respectively^12^.

**Figure 5:**
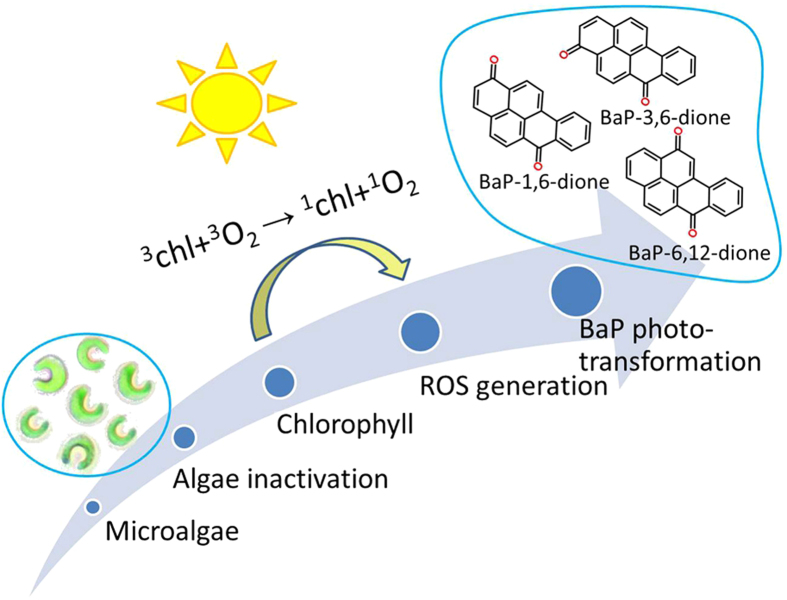
The mechanism of dead algal cells accelerating the photo-transformation of BaP. Chl, chlorophyll.

According to the changes of BaP metabolites shown in Fig. 4, the degradation pathway of BaP in live microalgal cells was different from that in dead cells. Although live and dead microalgae had the same chlorophyll, chlorophyll in live microalgae was protected from photo-degradation by carotenoids, and the reactive oxygen species (ROS) generated in live microalgae was scavenged by antioxidant defense systems^48^. Live microalgae metabolized BaP primarily via the dioxygenase pathway. In dead microalgae, the antioxidant defense systems were destroyed, BaP was photo-oxidized under the catalysis of chlorophyll molecules. The mechanism of dead algal cells in accelerating the photo-transformation of BaP was proposed as that chlorophyll in dead algal cells photo-oxidized BaP to quinones via photo-catalytic generation of singlet oxygen (Fig. 5).

No ring-fission product of BaP was detected in the treatments of dead algal cells and chlorophyll, dioxygenated BaP were the main products of BaP. However, the rate limiting steps of HMW PAH degradation are the introduction of molecular oxygen into aromatic ring since studies have shown greater PAHs degradation after partial oxidation^49^,^50^. Photo-transformation of BaP by chlorophyll could be considered as an initial step to increase BaP conversion to more susceptible intermediates for further degradation and mineralization by microorganisms.

The present study together with previous reports evidently demonstrated that chlorophyll accelerated the photo-transformation of BaP, which should be more applicable to wastewater treatment. In the natural environments, especially in algal blooms^51^, there is plenty of chlorophyll, which might contribute significantly to the clearance of organic contaminants, thus converting the harmful effect of algal blooms into environmental benefit. This study provides insightful information on the role of chlorophyll in the photo-transformation of toxic organic contaminants and renders a possible remediation strategy of organic pollutants in the environments.

## Materials and Methods

### Chemicals

Standards of BaP (98%), m-terphenyl (99%), acetone (99.5%), methanol (≥99.9%), benzene (99.8%), phenol (98%) and furfuryl alcohol (FFA, 97.5%) were obtained from Sigma-Aldrich (St. Louis, MO, USA). Chlorophyll *a* (>96%) was purchased from Wako Pure Chemical Industries, Ltd. (Japan). Five metabolites of BaP, 1-hydroxybenzo[a]pyrene (1-OH-BaP, >96%), 3-hydroxybenzo[a]pyrene (3-OH-BaP, >99%), benzo[a]pyrene-*cis*-4,5-dihydrodiol (BaP-*cis*-4,5-diol, >99%), benzo[a]pyrene-1,6-dione (BaP-1,6-dione, >99%) and benzo[a]pyrene-6,12-dione (BaP-6,12-dione, >99%) were supplied by Middlewest Research Institute (NCI Chemical Resource, Kansas, MO, USA). Ethyl acetate (99.8%) was obtained from LabScan Asia Company Limited (Thailand). Hydrochloric acid (HCl, 36%), sodium hydroxide (NaOH, 98%), sodium chloride and anhydrous sodium sulfate were provided by Farce Chemical Supplies (China). High-purity water was taken from a Milli-Q water system (Millipore, Eschborn, Germany).

### Photoreaction procedure

The irradiation experiments were performed under white light irradiation, which is a broad-spectrum light source that resembles the solar spectrum and has a wide range of wavelengths between 310 and 750 nm. White light was provided by a cool white fluorescent lamp (Philips essential TL5 14 W/840) at a light intensity of 50 μmol photons s^−1^ m^−2^. A series of 250-mL conical flasks were prepared and 100 mL sample solution was added into each flask. The algal cell density was 3.5 × 10^6^ cell mL^−1^. Dead algal cells were obtained by autoclaving at 121 °C for 10 min. The flasks only with culture medium were used as the abiotic controls for monitoring any abiotic loss of BaP. The initial concentration of BaP was 100 μg L^−1^. The flasks were then shaken on a rotary shaker at 160 rpm in an environmental chamber at 22  ± 2 °C with a 16:8 h light/dark cycle. Triplicate flasks from each of treatments were retrieved at different time intervals, and the residual amounts of BaP in the media and the algal cells were determined.

### Degradation of BaP by live and dead microalgal cells

Three different freshwater microalgal species were used to examine their BaP degradation efficiency with live and dead cells. *S. capricornutum* and *C. vulgaris* were purchased from Carolina Biological Supply Company, Burlington, NC, USA. *Chlorella* sp. was a local isolate enriched from influent collected from a sewage treatment plant in Hong Kong. Three algal species were cultured in Bristol medium^52^. The algae were grown under axenic conditions in an environmental chamber illuminated with cool white fluorescent tubes at a light intensity of 50 μmol m^−2^ s^−1^ at room temperature (22 ± 2 °C) and a diurnal cycle of 16 h light and 8 h dark. Algal cultures were continuously aerated with 0.22-μm membrane filtered air through a mechanical air pump. At the mid to late exponential growth phase (5–7 days), cells were harvested by centrifugation at 9,000 g for 10 min at 4 °C and washed twice with sterile deionised water^53^. The flasks were incubated under above-described condition, and the samples were collected after 4 days. The experiments were repeated with dead cells.

### Photo-transformation of BaP in aqueous solution containing denatured algae of *S. capricornutum*

The denatured algal cells were prepared using different methods, heat-killing and freezing-thawing. Heat-killing was conducted at 121 °C for 10 min. Freezing-thawing was used to break the cells with ten cycles of freezing in a refrigerator of –80 °C and thawing in a water bath of 40 °C. After freezing-thawing, cell viability was checked using a fluorescence microscope at ×400 magnification. Viable cells illuminate red fluorescence at the wavelength of 450 nm.

To prepare the supernatant of dead cells, *S. capricornutum* cells autoclaved at 121 °C for 10 min were separated immediately from the medium by centrifugation at 6,000 g for 15 min, and the supernatant was then spiked with BaP. The amount of BaP remained in the medium was determined at 6 h, 1, 2 and 4 days, respectively.

### Influence of chlorophyll on BaP photo-transformation

Harvested cells of *S. capricornutum* were extracted with 90% ethanol for 3 h in the dark. The cell extract was centrifuged for 10 min at 6,000 g and the absorbance of the supernatant was measured at the wavelengths of 630, 647, 664 and 750 nm by a UV-vis spectrophotometer. The chlorophyll *a* concentration was calculated according to the method described by Huang and Cong^54^. The chlorophyll concentration used in the experiment was set accordingly to the algal cell density of 3.5 × 10^6^ cell mL^−1^. Since dead algal cells were prepared by autoclaving at 121 °C for 10 min, the structure of chlorophyll was changed, converting into phaeophytin^31^. The same way was processed with chlorophyll to simulate the chlorophyll in dead algal cells. The experiment with synthetic chlorophyll *a* was carried out for comparison, the concentration of which was the same as the algal extracted chlorophyll. Samples were collected at 6 h, 1, 4 and 7 days, respectively. In order to investigate the effect of chlorophyll on the photo-transformation of BaP, different concentrations of synthetic chlorophyll *a* (0.1, 0.5 and 1.0 μg mL^−1^) were used.

### BaP analysis

Algal cells were separated from the medium by centrifugation at 6,000 g for 10 min at 4 °C. The BaP in the medium and taken up by microalgal cells was extracted with ethyl acetate according to the methods described by Ke *et al.*^28^. The samples were analysed with an Agilent Technologies 7890 gas chromatograph (GC) equipped with 5975 mass spectrometer (MS). An HP-5MS fused silica capillary column coated with 5% phenylmethyl polysiloxane (30 m length, 0.25 mm i.d., 0.25 μm film thickness; J&W Scientific, Folsom, CA) was used. An Agilent auto liquid sampler was used for sample injection, and the injection volume was 1.0 μL. Helium was the carrier gas, with a constant flow rate of 1.0 mL min^−1^. The injection mode was splitless, and the injector and detector temperatures were 280 °C and 300 °C, respectively. The GC column temperature was programmed from 90 °C to 200 °C at the rate of 30 °C min^−1^, and then 200 °C to 300 °C at the rate of 20 °C min^−1^, hold at 300 °C for 6 min. The samples were analysed in the selected ion monitoring (SIM) mode. The limit of detection (LOD), defined as a signal of three times the noise level, was 2.81 μg L^−1^.

### Detection of •OH and ^1^O_2_

The photoproduction of ROS was determined in the solution of dead algal cells and chlorophyll extracted from *S. capricornutum*. Benzene was used to trap •OH generated in the aqueous solution and produce phenol, thereby the phenol concentration could represent the concentration of •OH^26^,^55^. Benzene in the concentration of 100 μmol L^−1^ was added to the solution. Phenol was extracted with ethyl acetate and detected using GC-MS. The instrument parameters and methods were same as BaP analysis, with an exception of temperature program that was from 50 °C to 150 °C at the rate of 10 °C min^−1^, then increasing to 300 °C at the rate of 30 °C min^−1^. The recovery of phenol was 97.5% and the LOD was 0.0047 μmol L^−1^. The abiotic loss of phenol was also determined, which was negligible (~ 2.7% in 7 days).

FFA was used to detect ^1^O_2_ generated in the sample solution. It was recommended as an efficient trapping agent for ^1^O_2_ determination in the natural waters, and approximately 90% of the ^1^O_2_ could be trapped by FFA^56^. The initial concentration of FFA in the aqueous solution was 100 μmol L^−1^. ^1^O_2_ concentrations can be determined by the loss of FFA. FFA was analysed using high pressure liquid chromatograph (HPLC, Agilent Technologies 1200) packed with UV-vis detector. The detection wavelength was 218 nm and the mobile phase was methanol and water (50:50, v/v) at a flow rate of 1.0 mL min^−1^ using 150 × 4.6 mm Agilent C_18_ column, and the inject volume was 20 μL. The recovery of FFA was 96.6%, with the LOD of 3.43 μmol L^−1^.

### Determination of BaP metabolites

The Thermo Scientific LC system consisted of an Accela 1250 pump, and an Accela autosampler. BaP metabolites were chromatographically separated using a Hypeisil GOLD column (100 mm × 2.1 mm, i.d.; 1.9 μm Particle Size, Thermo Scientific) with methanol as the mobile phase A and water as the mobile phase B at a flow rate of 300 μL min^−1^. The linear gradient program was run as stated: 0 min, 75% (A); 4 min 90% (A); 6 min, isocratic of A 90%. The column temperature was kept at 25 °C. The detection was performed using Thermo Scientific TSQ Quantum Ultra mass spectrometer equipped with an atmospheric pressure chemical ionization (APCI) source. The measurements were performed in a positive ion mode at 400 °C vaporizer temperature, 350 °C capillary temperature, 40 psig sheath gas pressure and 8 psig aux gas pressure. The discharge current was set at 8.0 μA. The mass spectrometer was operated under select reaction monitoring (SRM) mode. The monitoring ion transitions, collision energy and retention time of BaP metabolites standards were shown in Supplementary Table 2.

### Statistical analysis

The mean and standard deviation values of triplicates were calculated. The effect of exposure time for chlorophyll *a* concentration was tested by one-way analysis of variance (ANOVA). If the ANOVA results were significant at *p* ≤ 0.05, Tukey’s multiple comparisons as post-hoc tests were applied to determine where the differences occured. All statistical analyses were carried out by a PC-compatible software package called SPSS (Version 16.0, SPSS Inc., Illinois, USA).

## Additional Information

**How to cite this article**: Luo, L. *et al.* Chlorophyll catalyse the photo-transformation of carcinogenic benzo[a]pyrene in water. *Sci. Rep.* **5**, 12776; doi: 10.1038/srep12776 (2015).

## Supplementary Material

Supplementary Information
