# Supplementary material for: Chlorophyll catalyse the photo-transformation of carcinogenic benzo[a]pyrene in water
Source: Sci Rep. 2015 Aug 4;5:12776. doi: 10.1038/srep12776 (PMC4523946; doi:10.1038/srep12776)
Supplement: Supplementary Information [file srep12776-s1.doc]

Supplementary Information

**Chlorophyll catalyse the photo-transformation of carcinogenic benzo[a]pyrene in water**

Lijuan Luo^1^, Xueying Lai^1^, Baowei Chen^1^, Li Lin^1^, Ling Fang^2^, Nora F. Y., Tam^3^, Tiangang Luan^1,*^

^1^MOE Key Laboratory of Aquatic Product Safety, School of Life Sciences/School of Marine Science, Sun Yat-sen University, Guangzhou 510275, China

^2^Instrumental Analysis and Research Center, Sun Yat-Sen University, Guangzhou 510275, China

^3^Department of Biology and Chemistry, City University of Hong Kong, Tat Chee Avenue, Kowloon, Hong Kong SAR, China

^*^Corresponding author

Prof. Tiangang Luan

Telephone: +86-20-84112958; Fax: +86-20-84037549

Email address: cesltg@mail.sysu.edu.cn (T. Luan)

**Supplementary Table 1. Retention times, selected ions and collision energy of the standards of BaP metabolites**

| Name | RT (min) | Mass ion peaks (*m/z*) | Collision Energy (ev) | With standard | Present or not |
| --- | --- | --- | --- | --- | --- |
| BaP-cis-4,5-diol | 1.81 | 269, 268, 252, 239^2^ | 47 | Yes | Yes |
| BaP-1,6-dione | 2.91 | 283, 255, 227, 226 | 43 | Yes | Yes |
| BaP-3,6-dione | 3.05 | 283, 255, 227, 226 | 43 | No | Yes |
| BaP-6,12-dione | 3.48 | 283, 255, 227, 226 | 43 | Yes | Yes |
| 1-OH-BaP | 6.25^1^ | 269, 268, 252, 239 | 47 | Yes | No |
| 3-OH-BaP | 6.25^1^ | 269, 268, 252, 239 | 47 | Yes | No |

^1^1-OH-BaP and 3-OH-BaP could not be separated.

^2^Underlined ion mass indicating the most abundant one among the ions.

**Supplementary Table 2.** ^1^O_2_ and •OH generation rates, BaP photo-transformation rates in dead *S. capricornutum*, chlorophyll and BaP solution.

|  | Dead *S. capricornutum* | Chlorophyll^1^ | BaP^2^ |
| --- | --- | --- | --- |
| ^1^O_2_ generation rate (µmol L^-1^ d^-1^) | 13.41 | 11.67 | 3.62 |
| •OH generation rate (µmol L^-1^ d^-1^) | 0.043 | 0.0015 | 0.0021 |
| BaP photo-transformation rate (d^-1^) | 1.187 | 0.832 | 0.018 |

^1^The chlorophyll was extracted from *S. capricornutum* and of which the concentration of chlorophyll *a* was 1.1 µg mL^-1^.

^2^BaP in blank algal culture medium of Bristol medium with the concentration of 100 µg L^-1^.

**Supplementary Fig. 1.** BaP photo-transformation under white light irradiation at different concentrations of chlorophyll (mean ± SD, n=3). Different letters on top of the bar of the same chlorophyll *a* concentration indicate the means are significantly different among the exposure time at the level of *p* ≤ 0.05.
